# Head-to-head comparison of non-invasive markers of atrial cardiomyopathy and their association with arrhythmia recurrence after atrial fibrillation ablation

**DOI:** 10.1007/s00392-026-02908-4

**Published:** 2026-03-30

**Authors:** Laura Dippel, Denis Fedorov, Julian Müller, Amir Jadidi, Dirk Westermann, Heiko Lehrmann, Thomas Arentz, Martin Eichenlaub

**Affiliations:** 1 Department of Cardiology and Angiology, University Heart Center Freiburg-Bad Krozingen, Suedring 15, 79189 Bad Krozingen, Germany; 2 Peter Osypka Institute for Medical Technology, Offenburg University of Applied Sciences, Badstrasse 24, 77652 Offenburg, Germany; 3 Arrhythmia and Electrophysiology Section, Heart Center Lucerne, Lucerne Cantonal Hospital, Spitalstrasse 31, 6000 Lucerne, Switzerland

**Keywords:** Atrial fibrillation, Atrial cardiomyopathy, Non-invasive markers, Electrocardiogram, Pulmonary vein isolation, Arrhythmia recurrence

## Abstract

**Background:**

Atrial cardiomyopathy (AtCM) represents an important substrate underlying atrial fibrillation (AF), increased arrhythmia recurrence after catheter ablation, and other adverse outcomes. Several non-invasive markers have been proposed as surrogates of AtCM, but their comparative performance and clinical relevance remain insufficiently validated.

**Methods:**

In this retrospective study, 200 patients undergoing first-time catheter ablation for symptomatic AF were included. All patients underwent high-density left atrial electroanatomical mapping, which served as reference standard for AtCM assessment based on the extent of left atrial low-voltage substrate (LA-LVS). Non-invasive AtCM markers derived from 12-lead electrocardiography (ECG), transthoracic echocardiography, and blood-based biomarkers were systematically compared with LA-LVS extent and their predictive value for arrhythmia recurrence during follow-up was assessed.

**Results:**

Among non-invasive AtCM markers, amplified P-wave duration (PWD) and P-wave amplitude in lead I showed the strongest association with LA-LVS extent. In multivariable logistic regression analysis, prolonged amplified PWD (≥ 150 ms) was independently associated with relevant AtCM (odds ratio 11.46, 95% confidence interval 2.27–57.90, *p* = 0.003). During a median follow-up of 277 days, arrhythmia recurrence occurred in 21.9% of patients. In Cox regression analysis, amplified PWD ≥ 150 ms was the only non-invasive AtCM marker independently associated with arrhythmia recurrence (hazard ratio 2.01, 95% confidence interval 1.07–3.78, *p *= 0.031).

**Conclusion:**

In patients undergoing first-time AF ablation, amplified PWD emerged as the most robust non-invasive marker of AtCM, independently associated with invasively assessed LA-LVS and arrhythmia recurrence. Advanced surface ECG analysis may represent a practical and widely applicable tool for AtCM-associated risk stratification in routine clinical practice.

**Graphical abstract:**

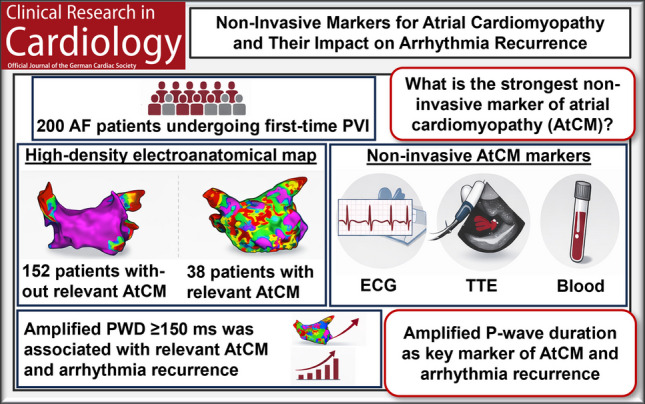

**Supplementary Information:**

The online version contains supplementary material available at 10.1007/s00392-026-02908-4.

## Introduction

Atrial cardiomyopathy (AtCM) has emerged as a key pathophysiological substrate underlying atrial fibrillation (AF) [1, 2]. Beyond increasing incidence of new-onset AF, AtCM promotes progression from paroxysmal to persistent AF and is associated with an increased risk of arrhythmia recurrence after pulmonary vein isolation (PVI) [1, 3–5]. Moreover, growing evidence suggests that AtCM also elevates stroke risk even in the absence of documented AF [1, 6, 7]. Consequently, AtCM has been recognized as a distinct pathological entity in two recently published consensus statements [8, 9].

At present, AtCM diagnosis primarily relies on invasive electroanatomical mapping (EAM) to identify left atrial low-voltage substrate (LA-LVS), limiting its applicability for preprocedural risk stratification [8–10]. Several non-invasive diagnostic approaches, including 12-lead electrocardiography (ECG), transthoracic echocardiography (TTE), and blood-based biomarkers, have been proposed as potential surrogates of AtCM [8, 9]. However, direct head-to-head comparisons of these markers with invasively quantified LA-LVS and systematic validation against clinically relevant outcomes remain limited, precluding their widespread adoption in routine clinical practice.

This study aimed to systematically compare non-invasive markers of AtCM with invasively quantified LA-LVS in a large cohort of AF patients undergoing first-time PVI and to evaluate their predictive value for arrhythmia recurrence following catheter ablation.

## Methods

This retrospective study included 200 patients with symptomatic AF who underwent first-time PVI including EAM at the Department of Cardiology and Angiology, Medical Center of the University of Freiburg.

Inclusion criteria comprised the availability of a digital 12-lead ECG and TTE in sinus rhythm prior to PVI, as well as a complete EAM acquired during sinus rhythm. Patients were excluded in the absence of a high-quality EAM or in case of any prior interventions in the left atrium.

Clinical characteristics, ECG, TTE, and electrophysiological data were obtained from multiple sources, including electronic medical records, the ECG database, TTE measurements, laboratory results, and the EAM system.

The study was performed in accordance with the Declaration of Helsinki and approved by the local ethics committee of the Medical Center of the University of Freiburg.

### P-wave analysis from 12-lead ECG

Standard 12-lead ECGs in sinus rhythm were digitally obtained using Cardiovit FT-1 (Schiller AG, Baar, Switzerland). Non-amplified P-wave duration (PWD) and P-wave axis were automatically computed by SEMA Software version 19.10 (Schiller AG, Baar, Switzerland). A non-amplified PWD ≥ 120 ms was considered prolonged, and a pathological P-wave axis was defined as < 0° or >  + 75°, in accordance with prior definitions [9, 11].

Analysis of amplified PWD was performed as previously described by our group [3, 12, 13]. In brief, unfiltered raw ECG data were used to generate an averaged beat for each lead, thereby reducing beat-to-beat P-wave variability and enhancing signal-to-noise ratio. Measurements were subsequently performed after ECG signal amplification (sweep speed: 175 mm/s; gain: 80 mm/mV) to enable optimal visualization of late, low-amplitude P-wave components. Amplified PWD was defined as the interval from the earliest onset of the P-wave in any lead to the latest offset in any lead, and a duration ≥ 150 ms was considered pathologically prolonged [9, 12]. All amplified PWD measurements were performed independently by two trained investigators who were blinded to any baseline characteristics and outcome data.

P-wave terminal force in lead V1 was calculated as the product of the amplitude (mV) and duration (ms) of the terminal negative component of the biphasic P-wave in lead V1, with values > 4 mV·ms considered pathological [9, 11]. P-wave amplitude was defined as the maximal P-wave voltage in lead I, with values ≤ 0.1 mV classified as pathological; P-wave dispersion was calculated as the difference between the longest and shortest PWD across all 12 leads, and values > 40 ms were considered pathological [9, 11, 14].

### TTE and blood-based biomarkers

TTE was performed in accordance with current guidelines from the American Society of Echocardiography and the European Association of Cardiovascular Imaging [15]. Left atrial diameter was measured at end-systole in the parasternal long-axis view. Left atrial volume index (LAVI) was calculated from apical four- and two-chamber views using Simpson’s biplane method and indexed to body surface area. Based on prior studies, LAVI thresholds of > 40 mL/m^2^, with > 48 mL/m^2^ for women aged > 65 years, were applied as pathological TTE markers of AtCM [9, 16].

Left ventricular ejection fraction (LV-EF) was determined using Simpson’s biplane method. In addition, presence of at least moderate mitral valve regurgitation was assessed.

For blood-based biomarkers, high-sensitivity C-reactive protein and high-sensitivity troponin T were measured from the baseline blood sample obtained at study inclusion.

### Voltage and activation mapping

High-density voltage and activation maps of the left atrium were acquired prior to PVI during sinus rhythm using a PentaRay catheter (electrode size: 1 mm, spacing: 2–6–2 mm, Biosense Webster, Irvine, CA, USA), in conjunction with the CARTO-3 EAM system as described previously [5, 17, 18]. Tissue proximity index was used in most of the cases to ensure catheter-tissue contact at each mapping point. In cases in which tissue proximity index was not used, LA-LVS was verified using a contact force–sensing ablation catheter (minimum contact force ≥ 5 g). This step was particularly relevant in cases of marked left atrial enlargement, where inadequate electrode–tissue contact might falsely suggest LA-LVS. The pulmonary veins and the mitral valve annuli were manually excluded prior to further analysis. The junction between the left atrium and the pulmonary veins was defined as 5 mm circumferential region proximal to the pulmonary vein ostia [19]. The mitral valve annulus was delineated based on local electrograms, identified by an atrial-to-ventricular signal amplitude ratio of approximately 1:2 [20].

Interatrial activation time (IAAT) was determined by measuring the interval between the earliest onset of the P-wave on surface ECG and the latest activated site of the left atrium (usually lateral left atrial wall below the left superior pulmonary vein or within the left atrial appendage) [17]. Patients with coronary sinus pacing (cycle length between 600–700 ms) during EAM were excluded from analysis of IAAT.

In accordance with the current AtCM consensus statement, LA-LVS was defined as areas with bipolar voltage amplitudes < 0.5 mV and quantified automatically using CartoNet (Biosense Webster, Irvine, CA, USA) [8]. LA-LVS extent served as reference standard for AtCM diagnosis and was calculated as ratio of the absolute surface area with LA-LVS and the total left atrial surface after exclusion of the pulmonary veins and the mitral valve annulus. Based on percentage of LA-LVS extent, AtCM was classified into four stages: stage I: < 5% (minimal), stage II: ≥ 5– < 20% (mild), stage III: ≥ 20– < 30% (moderate), and stage IV: ≥ 30% (extensive). Relevant AtCM was defined as LA-LVS extent ≥ 20% (stages III and IV) as described previously [10].

### Ablation procedure

Following EAM, wide antral circumferential PVI was performed using a contact force–sensing, irrigated-tip radiofrequency ablation catheter (Thermocool SmartTouch, tip electrode: 3.5 mm, spacing: 2–5–2 mm, Biosense Webster, Irvine, CA, USA). Target ablation index values were set at 400 for the posterior wall and 550 for the anterior wall, maintaining an inter-lesion distance of less than 5 mm [21]. Acute procedural success was defined as confirmed bidirectional electrical isolation of all pulmonary veins. In presence of documented or inducible macro-reentrant tachycardias involving the left atrium, further substrate modification was undertaken at the operator’s discretion. The specific type and extent of additional ablation sets were systematically recorded.

### Follow-up

Arrhythmia recurrence was assessed within a predefined 3- to 15-months follow-up window after PVI and was defined as any documented episode of AF, atypical atrial flutter, or left atrial tachycardia documented on ambulatory Holter-ECG (> 30 s lasting episode) or standard 12-lead ECG which was ascertained during a structured telephone interview using a standardized questionnaire [2].

### Objectives

The primary objective was to compare different non-invasive markers of AtCM in a large cohort of patients with AF undergoing first PVI with EAM. Secondary objective was to assess the predictive value of these AtCM markers for arrhythmia recurrence following PVI.

### Statistical analysis

Statistical analysis was performed using SPSS Statistics 29 (IBM, New York, NY, USA) and R version 4.5.2. Categorical variables are presented as number and percentage and were compared using Chi-square test. Continuous variables were assessed for normality using the Shapiro–Wilk test. Skewed variables are given as median with interquartile range (1st and 3rd quartiles) and analysed with Kruskal–Wallis tests, and normally distributed variables are presented as mean ± standard deviation and were compared with one-way ANOVA. Intraclass correlation coefficient estimates and their 95% confidence intervals (CI) for amplified PWD measurements by two independent investigators were calculated on the basis of a two-way mixed-effects model for consistency.

Univariable and multivariable logistic regression analyses were performed to identify variables associated with relevant AtCM (defined as LA-LVS extent ≥ 20%). Variables showing a statistically significant association with relevant AtCM in univariable logistic regression analyses (*p* < 0.05) were subsequently included in the multivariable model. In presence of collinearity, representative variables with the strongest clinical and statistical relevance were selected to avoid multicollinearity. Regression coefficients are reported as odds ratios (OR) with corresponding 95% CI. Continuous variables were standardized to one standard deviation (SD) for comparability.

Impact of clinical risk factors on arrhythmia recurrence was analysed using cox proportional hazards regression models. Kaplan–Meier curves were used to illustrate freedom from arrhythmia recurrence and compared using log-rank test. A two-tailed *P* < 0.05 was considered significant.

## Results

### Patient characteristics and extent of AtCM

A total of 200 patients (median age 69 [62–75] years, 70% male) undergoing first-time PVI were included. Baseline characteristics and procedural findings are summarized in Table 1.

**Table 1:** Baseline characteristics and procedural findings

Variables	Total cohort (*n* = 200)	AtCM stage I (*n* = 93)	AtCM stage II (*n* = 69)	AtCM stage III (*n* = 15)	AtCM stage IV (*n* = 23)	*P* value
Age, years	69 (62–75)	64 (60–71)	71 (64–76)	76 (72–80)	72 (68–80)	** < 0.001**
Male sex, n (%)	140 (70.0)	79 (84.9)	45 (65.2)	9 (60.0)	7 (30.4)	** < 0.001**
Female sex, n (%)	60 (30.0)	14 (15.1)	24 (34.8)	6 (40.0)	16 (69.6)	** < 0.001**
Body mass index, kg/m^2^	27.0 (24.8–30.8)	27.0 (24.9–29.5)	27.0 (24.3–31.6)	27.8 (25.6–30.6)	25.7 (20.8–31.2)	0.505
Paroxysmal AF, n (%)	91 (45.5)	46 (49.5)	31 (44.9)	5 (33.3)	9 (39.1)	0.600
Persistent AF, n (%)	109 (54.5)	47 (50.5)	38 (55.1)	10 (66.7)	14 (60.9)	0.600
Arterial hypertension, n (%)	134 (67.0)	56 (60.2)	48 (69.6)	13 (86.7)	17 (73.9)	0.154
Hypercholesterolemia, n (%)	81 (40.5)	36 (38.7)	34 (49.3)	4 (26.7)	7 (30.4)	0.213
Diabetes mellitus, n (%)	26 (13.0)	9 (9.7)	12 17.4)	2 (13.3)	3 (13.0)	0.555
Coronary artery disease, n (%)	34 (17.0)	12 (12.9)	16 (23.2)	2 (13.3)	4 (17.4)	0.373
Peripheral arterial disease, n (%)	7 (3.5)	0 (0.0)	4 (5.8)	0 (0.0)	3 (13.0)	**0.011**
Prior stroke/transient ischemic attack, n (%)	4 (2.0)	0 (0.0)	2 (2.9)	0 (0.0)	2 (8.7)	0.051
CHA₂DS₂-VA score	2 (1–3)	2 (1–2)	3 (1–4)	3 (2–4)	3 (2–4)	** < 0.001**
Beta-blocker, n (%)	111 (55.5)	55 (59.1)	39 (56.5)	4 (26.7)	13 (56.5)	0.134
Flecainide, n (%)	17 (8.5)	10 (10.8)	6 (8.7)	0 (0.0)	1 (4.3)	0.473
Class III antiarrhythmic drugs (amiodarone/dronedarone), n (%)	72 (36.0)	28 (30.1)	29 (42.0)	6 (40.0)	9 (39.1)	0.442
Sotalol, n (%)	4 (2.0)	2 (2.2)	1 (1.4)	0 (0.0)	1 (4.3)	0.784
Creatinine prior to PVI, mg/dL ^a^	0.99 (0.87–1.19)	1.0 (0.88–1.20)	0.96 (0.86–1.16)	1.01 (0.83–1.43)	0.94 (0.85–1.20)	0.758
LV-EF, % ^b^	62 (54–62)	62 (57–62)	62 (54–62)	57 (46–62)	57 (46–62)	**0.043**
Coronary sinus pacing, n (%)	81 (40.5)	34 (36.6)	35 (50.7)	3 (20.0)	9 (39.1)	0.161
IAAT, ms ^c^	125 (115–146)	121 (104–127)	125 (117–145)	145 (131–163)	166 (153–195)	** < 0.001**
LA-LVS extent (< 0.5 mV), %	5.8 (1.5–17.3)	1.1 (0.1–2.5)	9.5 (7.0–15.0)	23.6 (22.7–25.0)	42.0 (34.6–53.3)	** < 0.001**
PVI with additional left atrial ablation, n (%) ^d^	11 (5.5)	0 (0.0)	2 (2.9)	1 (6.7)	8 (34.8)	** < 0.001**

*AF* Atrial fibrillation, *AtCM* Atrial cardiomyopathy, *IAAT* Interatrial activation time, *LA-LVS* Left atrial low-voltage substrate, *LV-EF* Left ventricular ejection fraction, *PVI* Pulmonary vein isolation

^a^Available in 199/200 patients (92 in stage I, 69 in stage II, 15 in stage III and 23 in stage IV)

^b^Available in 192/200 patients (89 in stage I, 66 in stage II, 15 in stage III and 22 in stage IV)

^c^Available in 119/200 patients without coronary sinus pacing (59 in stage I, 34 in stage II, 12 in stage III and 14 in stage IV)

^d^Additional left atrial ablation was performed in a subset of patients, including roofline in seven patients, anterior mitral line in ten patients, septal mitral line in one patient and anterior box in one patient

Based on LA-LVS extent, 93 patients (46.5%) were classified as AtCM stage I (minimal), 69 patients (34.5%) as stage II (mild), 15 patients (7.5%) as stage III (moderate), and 23 patients (11.5%) as stage IV (extensive AtCM). Higher age and female sex were associated with more advanced AtCM stages. Median LA-LVS extent was 15.9 (5.4–34.2)% in women versus 3.8 (0.8–9.6)% in men (*p *< 0.001). In addition, peripheral arterial disease was more prevalent in advanced AtCM, and the CHA₂DS₂-VA score increased significantly with higher AtCM severity. Baseline medication use and renal function were comparable across AtCM stages. LV-EF was significantly lower in higher AtCM stages.

High-density EAM was performed with a median of 2,385 (1,624–3,803) points per left atrial map. In 35 patients (17.5%), tissue proximity index was not used during mapping (27 patients [29%] in AtCM stage I, 4 patients [6%] in stage II, 1 patient [7%] in stage III, and 3 patients [13%] in stage IV). Coronary sinus pacing was present in 81 patients (40.5%), with no significant difference in its distribution across AtCM stages. In the remaining 119 patients without coronary sinus pacing, a significant prolongation of IAAT was observed with increasing AtCM severity. As expected, patients with extensive LA-LVS more frequently underwent additional left atrial ablation beyond PVI.

### ECG, TTE, and blood-based markers of AtCM

Non-invasive biomarker findings are presented in Table 2.

**Table 2:** Non-invasive AtCM markers

Variables	Total cohort (*n* = 200)	AtCM stage I (*n* = 93)	AtCM stage II (*n* = 69)	AtCM stage III (*n* = 15)	AtCM stage IV (*n* = 23)	*P* value
Non-amplified PWD, ms	125 ± 21	124 ± 19	125 ± 18	124 ± 21	127 ± 36	0.957
Amplified PWD, ms	135 (125–146)	132 (124–140)	134 (124–146)	137 (131–150)	159 (153–168)	** < 0.001**
Pathological P-wave axis < 0° or > + 75°, n (%)	34 (17.0)	11 (11.8)	12 (17.4)	4 (26.7)	7 (30.4)	0.127
P-wave terminal force in lead V1, ms·mV	4.0 (2.6–6.5)	4.2 (2.7–7.0)	4.0 (2.6–6.2)	4.4 (3.1–6.1)	3.5 (1.8–4.7)	0.204
P-wave amplitude in lead I, mV	0.07 (0.06–0.10)	0.09 (0.06–0.10)	0.07 (0.05–0.09)	0.05 (0.04–0.07)	0.06 (0.04–0.08)	** < 0.001**
P-wave dispersion, ms	18 (10–28)	18 (12–29)	18 (11–27)	27 (8–31)	10 (8–24)	0.155
Left atrial diameter, mm ^a^	43 ± 6	42 ± 6	43 ± 6	45 ± 8	45 ± 6	0.079
LAVI, mL/m^2 b^	40 (31–50)	37 (30–45)	39 (30–48)	49 (39–57)	48 (42–60)	**0.002**
Relevant mitral regurgitation (≥ moderate), n (%) ^c^	13 (6.5)	1 (1.1)	6 (8.7)	0 (0.0)	6 (26.1)	** < 0.001**
High-sensitivity C-reactive protein prior to PVI, mg/L ^d^	1.4 (0.8–3.0)	1.0 (0.8–3.0)	1.9 (1.0–3.0)	1.0 (0.6–3.5)	1.0 (0.8–2.8)	0.479
High-sensitivity troponin T prior to PVI, ng/L ^e^	10 (7–14)	8 (6–11)	11 (8–16)	13 (11–17)	12 (9–17)	** < 0.001**

*AtCM* Atrial cardiomyopathy, *LAVI* Left atrial volume index, *PWD* P-wave duration

^a^ Available in 171/200 patients (81 in stage I, 60 in stage II, 10 in stage III and 20 in stage IV)

^b^ Available in 117/200 patients (54 in stage I, 36 in stage II, 11 in stage III and 16 in stage IV)

^c^ Available in 199/200 patients (92 in stage I, 69 in stage II, 15 in stage III and 23 in stage IV)

^d^ Available in 194/200 patients (89 in stage I, 69 in stage II, 13 in stage III and 23 in stage IV)

^e^ Available in 195/200 patients (90 in stage I, 69 in stage II, 14 in stage III and 22 in stage IV)

**Fig. 1:**
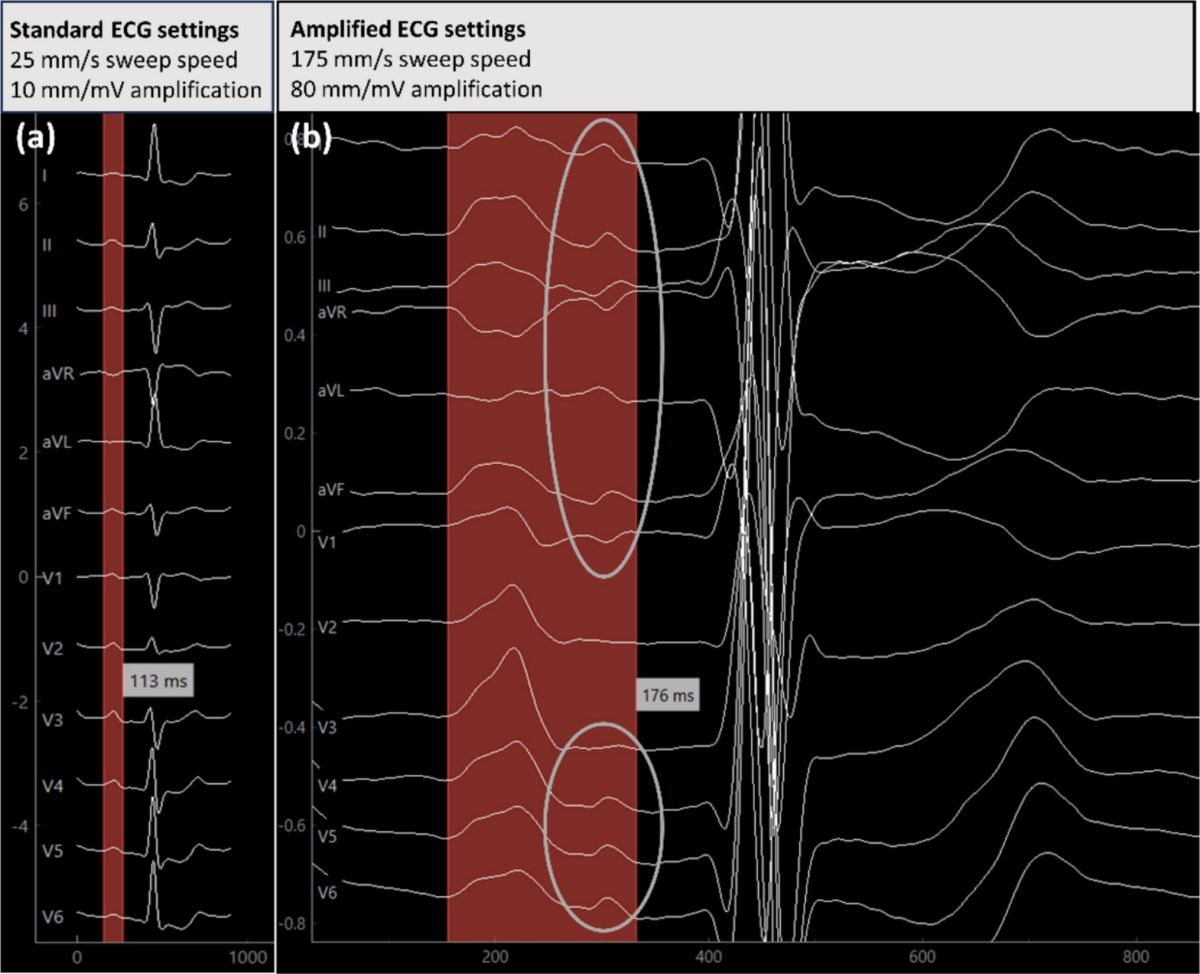
Visualization of PWD using standard and amplified ECG settings. Representative example of a patient with extensive left atrial low-voltage substrate (34%). The figure illustrates the difference between standard and amplified electrocardiography (ECG) settings for the assessment of P-wave duration (PWD). (**a**) Standard ECG settings (25 mm/s sweep speed, 10 mm/mV amplification). Using conventional ECG settings, non-amplified PWD appears relatively short (113 ms), as low-amplitude terminal atrial signals remain below the detection threshold. (**b**) Amplified ECG settings (175 mm/s sweep speed, 80 mm/mV signal amplification). Signal amplification reveals additional low-amplitude terminal P-wave components (grey circles), extending the measured amplified PWD to 176 ms, thereby uncovering the true prolonged atrial conduction time

While non-amplified PWD did not differ across AtCM stages, amplified PWD increased stepwise with advancing AtCM and was highest in patients with relevant AtCM (stages III–IV). A representative example illustrating the impact of ECG amplification on PWD measurement is shown in Fig. 1. Interobserver reliability for amplified PWD was high (intraclass correlation coefficient: 0.88, 95% CI: 0.81–0.89). P-wave amplitude in lead I decreased significantly with increasing AtCM severity.

TTE assessment revealed that LAVI was significantly higher in patients with relevant AtCM and the prevalence of at least moderate mitral regurgitation increased with AtCM severity.

Among blood-based biomarkers, high-sensitivity troponin T levels prior to PVI were significantly increased in patients with relevant AtCM.

### Correlation between non-invasive markers and invasive EAM parameters

**Fig. 2:**
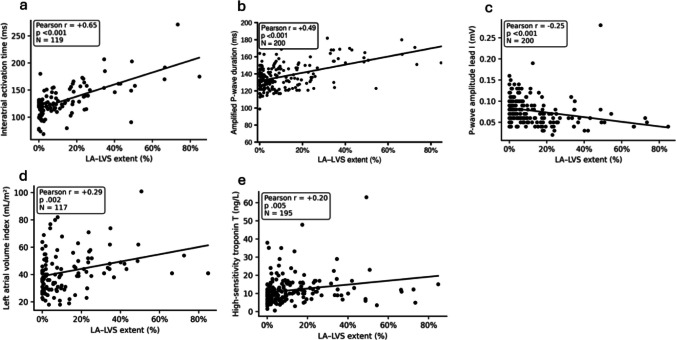
Correlation of invasive LA-LVS extent with non-invasive AtCM markers. Scatter plots illustrate the relationship between left atrial low-voltage substrate (LA-LVS) extent and (**a**) interatrial activation time, (**b**) amplified P-wave duration, (**c**) P-wave amplitude in lead I, (**d**) left atrial volume index, and (**e**) high-sensitivity troponin T. Pearson correlation coefficients (r), corresponding P-values, and sample sizes (N) are provided in each panel. Solid lines represent linear regression fits

IAAT was strongly correlated with LA–LVS extent (r = 0.65, *p *< 0.001, Fig. 2). Among ECG-derived biomarkers, amplified PWD showed significant correlations with both LA–LVS extent (r = 0.49,* p *< 0.001, Fig. 2) and invasive IAAT (r = 0.48, *p *< 0.001, Figure S1). Moreover, P-wave amplitude in lead I was inversely correlated with LA-LVS extent (r =  − 0.25, *p *< 0.001, Fig. 2) and IAAT (r =  − 0.27, *p *= 0.003, Figure S1).

With respect to TTE parameters, LAVI correlated with LA–LVS extent (r = 0.29, *p* = 0.002, Fig. 2) and IAAT (r = 0.41, *p *< 0.001, Figure S1).

Among blood-based biomarkers, high-sensitivity troponin T correlated with LA–LVS extent (r = 0.20, *p *= 0.005, Fig. 2) and IAAT (r = 0.32, *p* < 0.001, Figure S1).

### Predictors of relevant AtCM

In univariable logistic regression analyses (Table 3), older age, female sex, higher CHA₂DS₂-VA score, prolonged amplified PWD, pathological P-wave axis, lower P-wave amplitude in lead I, increased left atrial diameter and LAVI, reduced LV-EF, the presence of at least moderate mitral regurgitation, and elevated high-sensitivity troponin T levels were significantly associated with relevant AtCM and were therefore considered for multivariable modelling. Age and left atrial diameter were not included in the multivariable analysis because of collinearity with CHA₂DS₂-VA score and LAVI, respectively.

**Table 3:** Univariable regression analyses for presence of relevant AtCM

Variables	No relevant AtCM: LA-LVS < 20% (*n* = 162)	Relevant AtCM: LA-LVS ≥ 20% (*n* = 38)	Odds ratio per standard deviation (95% confidence interval)	*P* Value
Age, years	67 (60–73)	74 (70–80)	2.91 (1.86–4.81)	** < 0.001**
Female sex, n (%)	38 (23.5)	22 (57.9)	4.49 (2.14–9.40)	** < 0.001**
Body mass index, kg/m^2^	27.0 (24.8–30.9)	26.7 (24.6–30.7)	1.09 (0.76–1.51)	0.601
Persistent AF, n (%)	85 (52.5)	24 (63.2)	1.55 (0.76–3.28)	0.236
Arterial hypertension, n (%)	104 (64.2)	30 (78.9)	2.09 (0.94–5.17)	0.086
Hypercholesterolemia, n (%)	70 (43.2)	11 (28.9)	0.54 (0.25–1.15)	0.110
Diabetes mellitus, n (%)	21 (13.0)	5 (13.2)	1.02 (0.36–2.90)	0.974
Coronary artery disease, n (%)	28 (17.3)	6 (15.8)	0.90 (0.34–2.35)	0.825
Peripheral arterial disease, n (%)	4 (2.5)	3 (7.9)	3.39 (0.73–15.8)	0.121
Prior stroke/transient ischemic attack, n (%)	2 (1.2)	2 (5.3)	4.44 (0.61–32.61)	0.142
CHA₂DS₂-VA score	2 (1–3)	3 (2–4)	2.26 (1.50–3.41)	** < 0.001**
Beta-blocker, n (%)	94 (58.0)	17 (44.7)	0.59 (0.29–1.18)	0.140
Flecainide, n (%)	16 (9.9)	1 (2.6)	0.25 (0.03–1.92)	0.181
Class III antiarrhythmic drugs (amiodarone/dronedarone), n (%)	57 (35.2)	15 (39.5)	1.20 (0.58–2.48)	0.620
Sotalol, n (%)	3 (1.9)	1 (2.6)	1.43 (0.15–14.16)	0.759
Creatinine prior to PVI, mg/dL	0.99 (0.88–1.17)	0.97 (0.85–1.23)	0.99 (0.70–1.42)	0.975
Non-amplified PWD, ms	125 ± 19	126 ± 31	1.07 (0.75–1.52)	0.721
Prolonged non-amplified PWD ≥ 120 ms, n (%)	99 (61.5)	19 (50.0)	0.63 (0.31–1.28)	0.197
Amplified PWD, ms	132 (122–140)	152 (132–172)	3.50 (2.31–5.61)	** < 0.001**
Prolonged amplified PWD ≥ 150 ms, n (%)	17 (10.5)	24 (63.2)	14.62 (6.38–33.49)	** < 0.001**
Pathological P-wave axis < 0° or > + 75°, n (%)	23 (14.2)	11 (28.9)	2.46 (1.08–5.64)	**0.033**
P-wave terminal force in lead V1, mV·ms	4.1 (2.6–6.8)	3.6 (2.1–5.7)	0.90 (0.62–1.31)	0.592
Pathological P-wave terminal force in lead V1 > 4 mV·ms, n (%)	80 (49.4)	17 (44.7)	0.83 (0.41–1.69)	0.606
P-wave amplitude in lead I, mV	0.08 (0.06–0.10)	0.05 (0.04–0.07)	0.39 (0.22–0.67)	** < 0.001**
Pathological P-wave amplitude in lead I ≤ 0.1 mV, n (%)	133 (82.1)	36 (94.7)	3.93 (0.89–17.23)	0.070
P-wave dispersion, ms	19 (12–27)	11 (8–29)	0.87 (0.59–1.26)	0.452
Pathological P-wave dispersion > 40 ms, n (%)	13 (8.0)	3 (18.8)	1.04 (0.28–3.87)	0.951
Left atrial diameter, mm	42 ± 6	45 ± 6	1.51 (1.01–2.26)	**0.047**
LAVI, mL/m^2^	37 (30–46)	48 (41–57)	1.99 (1.27–3.11)	**0.003**
Pathological LAVI > 40 mL/m^2^, with > 48 mL/m^2^ for women aged > 65 years, n (%)	31 (34.4)	17 (63.0)	3.24 (1.32–7.91)	**0.010**
LV-EF, %	62 (55–62)	57 (46–62)	0.69 (0.50–0.97)	**0.032**
Relevant mitral regurgitation (≥ moderate), n (%)	7 (4.3)	6 (15.8)	4.13 (1.30–13.09)	**0.016**
High-sensitivity C-reactive protein prior to PVI, mg/L	1.5 (0.9–3.0)	1.0 (0.6–3.0)	0.86 (0.56–1.34)	0.513
High-sensitivity troponin T prior to PVI, ng/L	9 (7–13)	12 (9–17)	1.52 (1.10–2.10)	**0.011**

*AF* Atrial fibrillation, *AtCM* Atrial cardiomyopathy, *LA-LVS* Left atrial low-voltage substrate, *LAVI* Left atrial volume index, *LV-EF* Left ventricular ejection fraction, *PWD* P-wave duration, *PVI* pulmonary vein isolation

After multivariable adjustment using non-invasive AtCM markers as continuous variables (Table 4), female sex remained strongly and independently associated with relevant AtCM (OR: 22.55 [95% CI: 3.57–142.63], *p *< 0.001). Among non-invasive biomarkers, amplified PWD remained a significant predictor for relevant AtCM (OR per SD: 2.66 [95% CI: 1.23–5.75], *p* = 0.013), whereas higher P-wave amplitude in lead I was inversely associated (OR per SD: 0.20 [95% CI: 0.05–0.81], *p* = 0.024).

**Table 4:** Multivariate regression analysis for presence of relevant AtCM using non-invasive AtCM markers as continuous variables

Variables	Odds ratio per standard deviation	95% confidence interval	*P* value
Female sex	22.55	3.57–142.62	** < 0.001**
CHA₂DS₂-VA score	1.44	0.61–3.41	0.412
Amplified PWD, ms	2.66	1.23–5.75	**0.013**
Pathological P-wave axis	1.15	0.16–8.26	0.890
P-wave amplitude in lead I, mV	0.20	0.05–0.81	**0.024**
LAVI, mL/m^2^	1.02	0.97–1.07	0.359
LV-EF, %	0.65	0.25–1.68	0.369
Relevant mitral regurgitation (≥ moderate)	3.89	0.34–45.15	0.278
High-sensitivity troponin T prior to PVI, ng/L	1.60	0.56–4.61	0.383

*AtCM* Atrial cardiomyopathy, *LA-LVS* Left atrial low-voltage substrate, *LAVI* Left atrial volume index, *LV-EF* Left ventricular ejection fraction, *PWD* P-wave duration, *PVI* pulmonary vein isolation

When applying the predefined pathological thresholds for markers identified as significant in univariable analyses, female sex (OR: 20.31 [95% CI: 2.87–143.76], *p* = 0.003) and a prolonged amplified PWD ≥ 150 ms (OR: 11.46 [95% CI: 2.27–57.90], *p *= 0.003) were significantly associated with relevant AtCM (Table 5).

**Table 5:** Multivariate regression analysis for presence of relevant AtCM using pathological thresholds for non-invasive AtCM markers

Variables	Odds ratio per standard deviation	95% confidence interval	*P* value
Female sex	20.31	2.87–143.76	**0.003**
CHA₂DS₂-VA score	1.62	0.67–3.92	0.284
Prolonged amplified PWD ≥ 150 ms	11.46	2.27–57.90	**0.003**
Pathological P-wave axis	2.10	0.46–9.68	0.342
Pathological P-wave amplitude in lead I ≤ 0.1 mV ^a^			0.998
Pathological LAVI > 40 mL/m^2^, with > 48 mL/m^2^ for women aged > 65 years	2.29	0.37–14.22	0.374
LV-EF, %	0.44	0.16–1.19	0.103
Relevant mitral regurgitation (≥ moderate), n (%)	5.11	0.37–69.83	0.221
High-sensitivity troponin T prior to PVI, ng/L	1.21	0.48–3.03	0.679

*AtCM* Atrial cardiomyopathy, *LA-LVS* Left atrial low-voltage substrate, *LAVI* Left atrial volume index, *LV-EF* Left ventricular ejection fraction, *PWD* P-wave duration, *PVI* pulmonary vein isolation

^a^ This odds ratio could not be reliably estimated owing to model instability and is therefore not clinically interpretable

### Procedural characteristics and follow-up

Follow-up data were available for 192 of 200 patients (96%). During a median follow-up of 277 days (interquartile range 210–362 days), ECG-documented arrhythmia recurrence occurred in 42 patients (21.9%).

In Cox proportional hazards regression analysis, prolonged amplified PWD ≥ 150 ms (HR 2.01 [95% CI: 1.07–3.78], *p* = 0.031) was the only non-invasive marker independently associated with arrhythmia recurrence, in addition to the clinical CHA₂DS₂-VA score (HR 1.29 [95% CI: 1.02–1.62], *p* = 0.031) and presence of relevant AtCM defined by an LA-LVS extent ≥ 20% (HR 2.04 [95% CI: 1.05–3.94], *p* = 0.034).

**Fig. 3:**
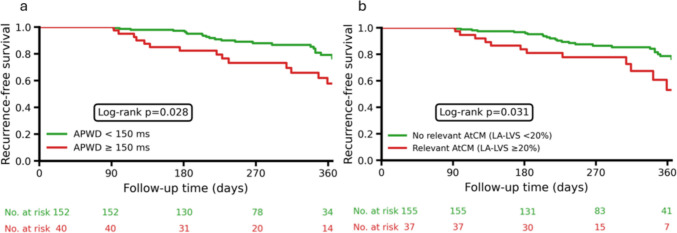
Arrhythmia-free survival after PVI. Kaplan–Meier curves illustrate arrhythmia-free survival during follow-up stratified by (**a**) amplified P-wave duration (APWD) < 150 ms versus ≥ 150 ms and (**b**) no relevant (left atrial low-voltage substrate [LA-LVS] extent < 20%) versus relevant atrial cardiomyopathy (AtCM, LA-LVS extent ≥ 20%). Log-rank P-values are shown in each panel and numbers of patients at risk for each group are indicated in the legend

As illustrated in Fig. 3, arrhythmia recurrence occurred more frequently in patients with amplified PWD ≥ 150 ms compared with those with amplified PWD < 150 ms (37.5% vs. 17.8%, *p* = 0.028), and in patients with relevant AtCM (LA-LVS extent ≥ 20%) compared with those without relevant AtCM (35.1% vs. 18.7%, *p* = 0.031).

## Discussion

**Fig. 4:**
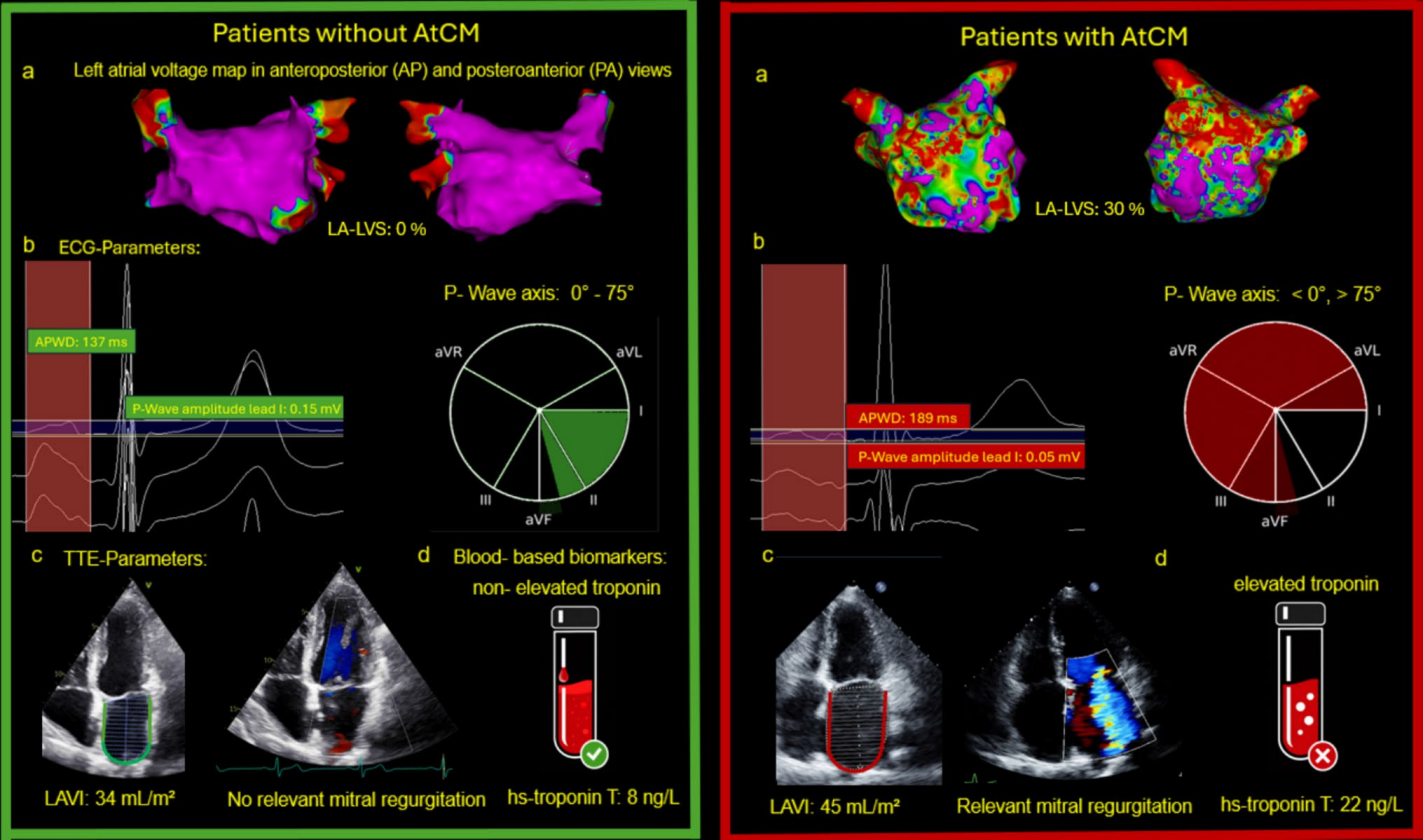
Multimodal characterization of AtCM**.** Representative examples of patients without AtCM (left panel, green frame) and with relevant AtCM (right panel, red frame) integrating invasive electroanatomical mapping (EAM), electrocardiography (ECG), transthoracic echocardiography (TTE), and blood-based biomarkers. (**a**) Electroanatomical voltage maps of the left atrium demonstrating preserved atrial voltage in patients without AtCM and extensive left-atrial low-voltage substrate (LA-LVS) in patients with relevant AtCM. (**b**) ECG analysis illustrating shorter amplified P-wave duration (APWD), higher P-wave amplitude in lead I and normal P-wave axis in patients without AtCM, compared with prolonged APWD, reduced P-wave amplitude in lead I and pathological P-wave axis in patients with relevant AtCM. (**c**) TTE showing normal left atrial volume index (LAVI) and absence of relevant mitral regurgitation in patients without AtCM versus left atrial enlargement and relevant mitral regurgitation in patients with relevant AtCM. (**d**) High-sensitivity troponin T as a blood-based biomarker was within the normal range in patients without AtCM and elevated in patients with relevant AtCM

In this comprehensive analysis of patients undergoing first-time AF ablation, we evaluated the clinical utility of non-invasive AtCM markers to identify advanced atrial disease and predict rhythm outcomes. Several key findings emerge: First, among a broad panel of ECG-, TTE-, and blood-based parameters, amplified PWD and P-wave amplitude in lead I demonstrated the strongest and most consistent associations with invasively quantified LA-LVS, which served as the reference standard for AtCM (Fig. 4). Second, amplified PWD was the only non-invasive AtCM marker that was not only associated with advanced atrial disease but also independently predicted arrhythmia recurrence following first-time PVI.

### EAM for AtCM quantification

The concept of AtCM describes atrial remodeling beyond the mere presence of AF, encompassing structural, electrical, and functional alterations of the atrial myocardium [8, 9, 13]. In this context, LA-LVS identified by EAM have emerged as the most established surrogate of AtCM and have been consistently linked to adverse ablation outcomes [10, 13]. The definition of LA-LVS based on a bipolar voltage threshold of < 0.5 mV represents a simplified but widely used approach for characterizing atrial structural remodeling and is therefore also adopted in the current AtCM consensus statement [8]. However, histological validation studies have demonstrated that normal bipolar voltage varies across different atrial regions and that atrial structural remodeling in patients with AF is not solely characterized by localized fibrosis but rather represents a diffuse process involving fibrosis, increased intercellular space, myofibrillar loss, and reduced cardiomyocyte density [4, 22, 23]. Consequently, it may not be appropriate to assume a single universal LA-LVS cut-off for accurately quantifying the extent of AtCM [4]. Nevertheless, while alternative approaches such as individualized voltage thresholds or analysis of global voltage distributions may provide additional insights, the commonly used threshold of < 0.5 mV remains a pragmatic and widely accepted definition for clinically relevant atrial substrate [4, 8, 22]. This threshold has therefore been applied in many studies investigating AtCM and AF ablation outcomes and was also used in our study to ensure comparability with previous literature and to facilitate interpretation within established AtCM staging frameworks.

Another important aspect relates to technical factors that may influence voltage mapping. Several studies have demonstrated that bipolar electrogram amplitudes can vary depending on the activation rate, the orientation of the activation wavefront relative to the electrode pair, and the inter-electrode spacing of the mapping catheter, potentially leading to differences in the detected extent of LA-LVS [24–26]. In our study, coronary sinus pacing during EAM was performed in 81 patients (40.5%), while in the remaining 119 patients mapping was conducted during sinus rhythm. A high-density multipolar mapping catheter with small inter-electrode spacing was used in all patients, which likely mitigates wavefront direction–dependent effects by sampling activation from multiple orientations and thereby improves the robustness of voltage characterization [27]. In addition, coronary sinus pacing was performed at cycle lengths between 600 and 700 ms, which approximate the intrinsic sinus cycle length in most patients and bipolar voltage amplitudes are therefore likely to remain largely comparable between sinus rhythm and coronary sinus pacing [24].

Yamaguchi et al. defined four stages of LA-LVS and demonstrated that patients with relevant LA-LVS (≥ 20% of the left atrial surface area) exhibited a significantly increased recurrence rate following PVI which is consistent with the findings in our study [10]. However, the invasive nature of EAM limits its applicability for preprocedural risk stratification [8, 9].

### Non-invasive ECG markers as surrogates of AtCM

Our findings demonstrate that amplified PWD closely reflects the extent of LA-LVS. This observation aligns with prior reports linking prolonged PWD to interatrial conduction abnormalities in patients with AtCM [12, 13]. By enhancing low-amplitude terminal P-wave components, amplified ECG analysis appears particularly sensitive to advanced atrial remodeling and may therefore outperform standard non-amplified PWD in detecting conduction delay [12]. This mechanism may also explain observations from earlier studies in which both prolonged and paradoxically short non-amplified PWD were associated with an increased risk of AF and stroke [28, 29]. In individuals with advanced AtCM, delayed terminal atrial activation may remain undetected on standard ECG recordings, resulting in an apparently short PWD despite prolonged overall atrial conduction. Signal amplification enables visualization of these terminal components and thus provides a more accurate estimate of total atrial activation time. Consistent with this concept, amplified PWD showed a strong correlation with IAAT in our cohort, supporting its pathophysiological relevance as a marker of global atrial conduction delay. Moreover, the predefined cut-off of ≥ 150 ms was associated with a more than 11-fold increased risk of relevant AtCM in multivariable regression analysis and emerged as the only AtCM marker significantly associated with arrhythmia recurrence, corresponding to an approximately twofold increased risk after PVI [12].

In parallel, reduced P-wave amplitude in lead I was an independent marker of relevant AtCM [11]. Decreased atrial P-wave amplitude likely reflects loss of viable atrial myocardium, altered atrial activation vectors, or electrical uncoupling due to fibrotic remodeling [11, 14]. While P-wave amplitude has received less attention than PWD, our results suggest that it captures complementary information on atrial myocardial integrity and may therefore enhance non-invasive substrate characterization. However, the predefined cut-off of ≤ 0.1 mV was not significantly associated with relevant AtCM in our cohort, and, in contrast to amplified PWD, P-wave amplitude did not predict arrhythmia recurrence following PVI.

### Comparison with TTE and blood-based biomarkers

Elevated LAVI, reduced LV-EF, relevant mitral regurgitation, and increased high-sensitivity troponin T levels were all associated with relevant AtCM in univariable analyses, reflecting the close interplay between atrial enlargement, ventricular function, and myocardial injury [8, 9, 16]. However, none of these parameters retained independent significance after adjustment for ECG-derived markers and other clinical risk factors.

These findings suggest that while these TTE and blood-based biomarkers reflect global cardiovascular disease burden, ECG markers may more directly capture atrial-specific electrical remodeling, particularly compared with structural markers of atrial dilatation alone. However, left atrial strain analysis, which represents a more specific marker of AtCM by directly quantifying atrial mechanical function, was not available in the present study [5].

### Sex-specific aspects of AtCM

Female sex emerged as a strong independent predictor of relevant AtCM and female patients exhibited a substantially higher burden of LA-LVS compared with men (15.9% versus 3.8%, *p* < 0.001) in the present study. This finding is consistent with previous reports demonstrating more advanced AtCM, including more extensive LA-LVS, lower global atrial voltage, slower conduction velocity and a higher proportion of complex fractionated signals, as well as worse ablation outcomes in women despite smaller atrial dimensions [30–32]. Potential underlying mechanisms include hormonal influences on fibrotic remodelling and later referral for catheter ablation procedures [33, 34]. However, the interpretation of sex-related differences in LA-LVS is complex. Recent studies combining high-density EAM with atrial biopsy have demonstrated that women exhibit lower atrial voltage even in the absence of AF, which may partly reflect intrinsic differences in atrial myocardial architecture, including smaller cardiomyocyte size and lower atrial mass [35]. Consequently, the use of uniform bipolar voltage thresholds could potentially lead to differences in the detected extent of LA-LVS between sexes. Although the present study was not designed to investigate mechanistic sex differences, our findings underscore the importance of incorporating sex-specific considerations into AtCM assessment and risk stratification. Notably, despite a greater LA-LVS extent, arrhythmia recurrence rates did not differ significantly between men and women in our cohort. Therefore, sex-specific voltage thresholds for defining LA-LVS may deserve consideration in future studies.

### Clinical implications

To the best of our knowledge, this is the first study to systematically compare non-invasive AtCM markers in a large cohort of patients with AF using comprehensive EAM data as a reference standard for AtCM assessment (Fig. 4). Our findings align with recent consensus statements emphasizing electrical and structural atrial remodelling as core components of AtCM and have several clinically relevant implications [8, 9].

First, ECG-derived markers, most notably amplified PWD, enable non-invasive identification of patients with advanced AtCM prior to ablation. This may improve procedural planning, for example by supporting the decision to perform PVI in conjunction with EAM to identify and treat additional extra-pulmonary vein substrate, as was applied in patients with more extensive LA-LVS in the present cohort. Second, the association between amplified PWD and arrhythmia recurrence suggests that ECG-based AtCM markers capture atrial substrate characteristics that extend beyond pulmonary vein triggers and contribute to ablation failure as shown in our cohort. This information may be valuable for pre-procedural risk communication and expectation management prior to PVI. Moreover, accumulating evidence indicates that AtCM is independently associated with an increased risk of thromboembolic events, even in the absence of documented AF [1, 6, 7]. Non-invasive markers of AtCM may therefore not only support rhythm-related risk stratification but could also contribute to improved assessment of stroke risk. However, this potential application requires validation in large, prospective outcome studies.

Importantly, surface ECG analysis appears particularly well suited for screening purposes. ECGs are inexpensive, ubiquitously available, and easily repeatable, and their interpretation does not rely on advanced imaging infrastructure or specialized TTE expertise. Signal amplification can be readily implemented using several standard digital ECG systems without the need for additional hardware, suggesting that amplified PWD may represent a simple and accessible tool for non-invasive identification of advanced atrial disease in clinical practice. Nevertheless, automated algorithms for amplified PWD measurement will be required to enable scalable implementation in routine practice. With such developments, ECG-based assessment of AtCM could represent a pragmatic first-line screening tool, particularly in settings with limited access to advanced imaging modalities.

### Limitations

Several limitations merit consideration. First, this was a single-center, retrospective study, which may limit generalizability. Second, although high-density EAM was performed using standardized protocols, voltage thresholds and mapping techniques remain subject to methodological variability. Nevertheless, a widely used bipolar LA-LVS cut-off (< 0.5 mV) was applied, and mapping quality was ensured by high point density and contact verification, supporting the robustness of LA-LVS quantification.

Third, tissue proximity index during EAM was not used in all procedures. In these cases, LA-LVS was verified using a contact force–sensing ablation catheter to confirm adequate PentaRay catheter–tissue contact. While larger electrode size of the ablation catheter limits spatial resolution compared with the multipolar mapping catheter, this verification step was primarily implemented to exclude false-positive LA-LVS due to insufficient tissue contact rather than to replace high-density mapping. Moreover, the majority of patients without tissue proximity index were classified as AtCM stage I, where the absence of substantial LA-LVS makes misclassification due to insufficient tissue contact unlikely. Fourth, arrhythmia recurrence was assessed using intermittent rhythm monitoring, which may underestimate asymptomatic recurrences. Nevertheless, this approach reflects routine clinical follow-up in many centers and is unlikely to differentially affect comparisons between marker-defined subgroups. Fifth, while the analysis focused on established ECG-derived parameters, more advanced signal-processing techniques or machine-learning–based approaches were not evaluated. Finally, inclusion required the availability of a baseline ECG recorded in sinus rhythm to enable AtCM assessment, which may have introduced selection bias. However, this criterion was necessary to ensure reliable AtCM marker analysis.

## Conclusion

In patients undergoing first-time PVI, amplified PWD emerged as the most robust non-invasive marker of relevant AtCM, independently associated with invasively assessed LA-LVS and arrhythmia recurrence. These findings support advanced surface ECG analysis as a practical and widely applicable tool for identifying advanced atrial disease and improving pre-procedural risk stratification in patients undergoing AF ablation.

## Supplementary Information

Below is the link to the electronic supplementary material.

ESM 1 (DOCX 135 KB)

## Data Availability

The datasets generated and analysed during the current study are available from the corresponding author on reasonable request.
