# Supplementary material for: Head-to-head comparison of non-invasive markers of atrial cardiomyopathy and their association with arrhythmia recurrence after atrial fibrillation ablation
Source: Clin Res Cardiol. 2026 Mar 30;115(6):1027–41. doi: 10.1007/s00392-026-02908-4 (PMC13160966; doi:10.1007/s00392-026-02908-4)
Supplement: Supplementary file 1 — (DOCX 135 KB) [file 392_2026_2908_MOESM1_ESM.docx]

**Supplementary Material, Clinical Research in Cardiology:**

**Head-to-Head** **Comparison of Non-Invasive Markers of Atrial Cardiomyopathy and Their Association With Arrhythmia Recurrence After Atrial Fibrillation Ablation**

Laura Dippel^1,2^, Denis Fedorov^1^, Heiko Lehrmann^1^, Julian Müller^1^, Amir Jadidi ^1,3^, Dirk Westermann^1^, Thomas Arentz^1^, Martin Eichenlaub^1^

^1^ Department of Cardiology and Angiology

University Heart Center Freiburg-Bad Krozingen,

Suedring 15, 79189 Bad Krozingen, Germany

^2^ Peter Osypka Institute for Medical Technology

Offenburg University of Applied Sciences

Badstrasse 24, 77652 Offenburg, Germany

^3^ Arrhythmia and Electrophysiology Section

Heart Center Lucerne

Lucerne Cantonal Hospital

Spitalstrasse 31, 6000 Lucerne, Switzerland

Corresponding author:

Dr. Martin Eichenlaub

martin.eichenlaub@uniklinik-freiburg.de

**Fig. S1** Correlation of invasive interatrial activation time with non-invasive AtCM markers


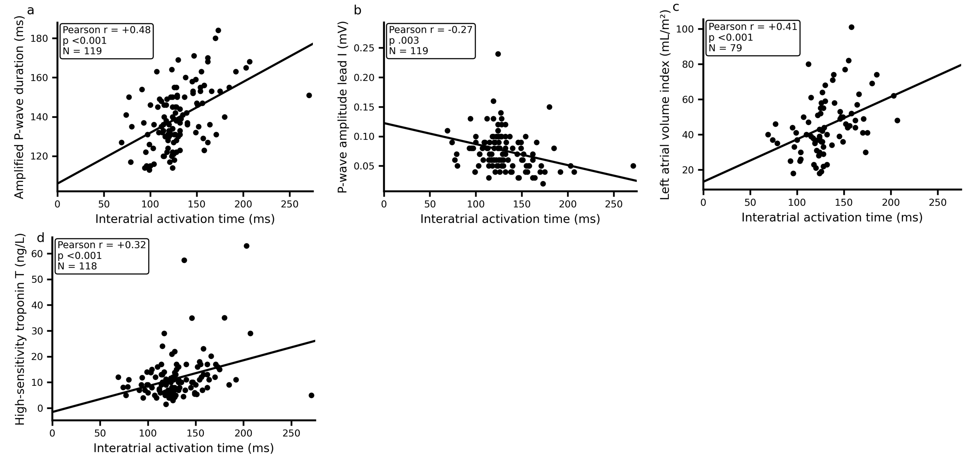


Scatter plots illustrate the relationship between interatrial activation time and (a) amplified P-wave duration, (b) P-wave amplitude in lead I, (c) left atrial volume index and (d) high-sensitivity troponin T. Pearson correlation coefficients (r), corresponding P-values, and sample sizes (N) are provided in each panel. Solid lines represent linear regression fits.
